# 63例HIV相关弥漫大B细胞淋巴瘤临床特征及预后分析：国内单中心真实世界研究

**DOI:** 10.3760/cma.j.issn.0253-2727.2022.03.004

**Published:** 2022-03

**Authors:** 超雨 王, 俊 刘, 喜平 梁, 冰凌 郭, 人之 胡, 耀 刘

**Affiliations:** 重庆大学附属肿瘤医院血液肿瘤中心，肿瘤转移与个体化诊治转化研究重庆市重点实验室，重庆 400030 Department of Hematology-Oncology, Chongqing University Cancer Hospital, Chongqing Key Laboratory of Translational Research for Cancer Metastasis and Individualized Treatment, Chongqing 400030, China

**Keywords:** 淋巴瘤，大B细胞，弥漫性, 人类免疫缺陷病毒, 获得性免疫缺陷综合征, 临床特征, 预后, Lymphoma, large B-cell, diffuse, Human immunodeficiency virus, Acquired immune deficiency syndrome, Clinical characteristics, Prognosis

## Abstract

**目的:**

探讨HIV相关弥漫大B细胞淋巴瘤（DLBCL）患者的临床特征及预后。

**方法:**

回顾性分析2008年7月至2021年8月重庆大学附属肿瘤医院确诊的63例HIV相关DLBCL患者的临床资料。生存分析采用Kaplan-Meier方法，组间生存的比较采用Log-rank检验，多因素分析采用Cox回归模型。

**结果:**

63例患者中，男57例（90.5％），中位年龄49（23～87）岁。主要病理类型为生发中心来源（GCB），为47例（74.6％）。29例（46.0％）患者合并淋巴结外病变，17例（27.0％）患者合并大包块（最大直径≥7.5 cm），20例（31.7％）患者伴随B症状。CD4^+^淋巴细胞中位数为203（4～1022）×10^6^/L，49％（25/51）的患者CD4^+^细胞数<200×10^6^/L，51％（26/51）的患者CD4^+^细胞数≥200×10^6^/L。43.1％（25/58）的患者IPI评分0～2分，56.9％（33/58）的患者IPI评分 3～5分。78％（46/59）的患者Ann Arbor分期Ⅲ/Ⅳ期。16例（25.4％）患者未接受化疗，14例（22.2％）患者接受小于4个周期的化疗，33例（52.4％）患者接受4个周期及以上的化疗。在接受化疗的患者中，61.7％（29/47）的患者采用R-CHOP样方案，38.3％（18/47）的患者采用CHOP样方案。总体患者的1、2、3和5年总生存（OS）率分别为65.0％、53.8％、47.1％和43.5％。单因素分析显示年龄≥60岁（*P*＝0.012）、美国东部肿瘤协作组体能状况（ECOG-PS）评分2～4分（*P*＝0.043）、IPI评分3～5分（*P*＝0.001）、β_2_-微球蛋白升高（*P*＝0.007）和全身化疗周期数<4（*P*<0.001）为影响患者OS的不良预后因素。Cox多因素分析显示年龄≥60岁（*HR*＝2.272，95％*CI* 1.110～4.651，*P*＝0.025）、IPI评分3～5分（*HR*＝3.562，95％*CI* 1.794～7.074，*P*<0.001），ECOG-PS评分2～4分（*HR*＝2.675，95％*CI* 1.162～6.153，*P*＝0.021）和化疗周期数<4（*HR*＝0.290，95％*CI* 0.176～0.479，*P*<0.001）为影响OS的预后危险因素。

**结论:**

HIV相关DLBCL是最常见的HIV相关肿瘤，男性多见，1年内病死率高，化疗联合抗逆转录病毒治疗可显著改善患者预后。

## Abstract (en)

**Objective:**

This study aimed to look into the clinical characteristics and prognosis of patients with human immunodeficiency virus（HIV）-associated diffuse large B-cell lymphoma（DLBCL）.

**Methods:**

Retrospective review of the clinical data of 63 HIV-infected patients with DLBCL diagnosed at Chongqing University Cancer Hospital between July 2008 and August 2021. The Kaplan-Meier method was used to calculate survival curves, and the log-rank test method was used to compare survival between groups. The Cox proportional hazards model was used for multivariate analysis.

**Results:**

In 63 patients with HIV-associated DLBCL, 57（90.5％）were men, and the median age was 49（23–87）years. The most common pathological subtype was the germinal center B-cell-like lymphoma（74.6％）; 46.0％（29/63）were combined with extranodal lesions. Seventeen of 63（27.0％）patients had large masses（≥7.5 cm）. Twenty of 63（31.7％）patients had B symptoms. The median CD4^+^ T cell count was 203（4–1022）×10^6^/L. A total of 49％（25/51）patients had CD4^+^ cell count <200×10^6^/L, 56.9％（33/58）had high（3–5）International Prognostic Index（IPI）scores, and 43.1%（25/58）had low（0–2）IPI scores. Further, 78％（46/59）were diagnosed with Ann Arbor Stage Ⅲ/Ⅳ, and 25.4％（16/63）didn't receive chemotherapy. A total of 22.2％（14/63）of patients received less than four cycles of chemotherapy, and 52.4％（33/63）received four or more cycles of chemotherapy. Among patients undergoing chemotherapy, 61.7％（29/47）received R-CHOP-like regimens, and 38.3％（18/47）used CHOP-like regimens. The 1-, 2-, 3-, and 5-year overall survival（OS）rates were 65.0％, 53.8％, 47.1％, and 43.5％, respectively. Univariate analysis revealed that age ≥ 60 years（*P*＝0.012）, Eastern Cooperative Oncology Gruop Performance Status（ECOG-PS）score 2–4 points（*P*＝0.043）, IPI score 3–5 points（*P*＝0.001）, β_2_-MG elevation（≥5.5 mg/L）（*P*＝0.007）, and systemic chemotherapy cycles less than four times（*P*<0.001）were the negative prognostic factors affecting the OS of patients. The Cox multivariate analysis depicted that age ≥60 years（*HR*＝2.272, 95％ *CI* 1.110–4.651, *P*＝0.025）, IPI score 3–5 points（*HR*＝3.562, 95％ *CI* 1.794–7.074, *P*<0.001）, ECOG-PS score 2–4 points（*HR*＝2.675, 95％ *CI* 1.162–6.153, *P*＝0.021）, and number of cycles of chemotherapy<4（*HR*＝0.290, 95％ *CI* 0.176–0.479, *P*<0.001）were independent risk factors for adverse prognosis of OS.

**Conclusion:**

HIV-associated DLBCL is the most common HIV-related tumor, is most commonly seen in men, and has a high 1-year mortality rate. Chemotherapy combined with antiretroviral therapy can improve patient prognosis.

世界卫生组织将人类免疫缺陷病毒（human immunodeficiency virus，HIV）感染相关肿瘤分为艾滋病定义肿瘤（AIDS-defining cancer，ADC）和非艾滋病定义肿瘤（non AIDS-defining cancer，NADC）。1993年美国疾病预防控制中心根据艾滋病患者的患癌风险, 将卡波西肉瘤（Kaposi's sarcoma，KS）、非霍奇金淋巴瘤（non-Hodgkin lymphoma，NHL）和浸润性宫颈癌（invasive cervical cancer，ICC）命名为ADC；NADC包括肝癌、霍奇金淋巴瘤、肛门癌、肺癌、皮肤癌、结肠直肠癌等[1]。2018年，美国国家癌症研究所最新的统计数据显示，在ADC中，NHL发病率超过KS，成为发病率最高的HIV感染相关肿瘤[2]。HIV相关弥漫大B细胞淋巴瘤（diffuse large B-cell lymphoma，DLBCL）是发病率最高的HIV相关NHL[3]，严重影响HIV患者的生存，但总体发病率较低、地区差异、对HIV患者存在偏见等因素制约了对HIV相关DLBCL的研究。我国关于HIV相关DLBCL的大宗病例报道较少，本研究回顾性分析了重庆大学附属肿瘤医院63例HIV相关DLBCL患者的临床特征及预后，以提高临床医师对此病的认识。

## 病例与方法

1. 病例：本研究回顾性分析重庆大学附属肿瘤医院血液肿瘤中心2008年7月至2021年8月诊断的63例HIV相关DLBCL患者的人口学资料、临床表现、实验室检查及治疗方案等，2013年9月前诊断患者2例，其余61例患者于2013年9月后诊断。

2. 诊断标准：DLBCL诊断参照中国弥漫大B细胞淋巴瘤诊断与治疗指南（2013年版）[4]，所有患者均合并HIV感染。

3. 疗效评估：疗效评估标准同样参照中国弥漫大B细胞淋巴瘤诊断与治疗指南（2013年版）[4]，分为完全缓解（CR）、部分缓解（PR）、疾病稳定（SD）和疾病进展（PD）。

4. 随访：通过查阅患者住院病历和电话随访的方式进行随访，随访截止日期为2021年10月31日，中位随访时间11（1～161）个月。无进展生存（PFS）时间定义为自DLBCL确诊至疾病进展、死亡或末次随访的时间间隔，总生存（OS）时间定义为自DLBCL确诊至患者死亡或末次随访的时间间隔。

5. 统计学处理：采用SPSS 25.0软件进行统计学分析，Graph PadPrism Version 9.0.0作图。连续变量采用中位数（范围）表示。计数资料用例数（百分比）表示。生存曲线采用Kaplan-Meier法绘制，OS和PFS采用Kaplan-Meier法进行分析，单因素生存分析采用Log-rank检验，多因素生存分析采用Cox回归模型，*P*<0.05为差异有统计学意义。

## 结果

1. 临床特征：63例HIV相关DLBCL患者中男57例（90.5％），女6例（9.5％），诊断时中位年龄49（23～87）岁，其中<60岁48例（71.2％）。HIV感染途径：异性性行为19例（30.2％），同性性行为2例（3.2％），血液接触2例（3.2％），其他40例（63.5％）。25例（39.7％）患者确诊淋巴瘤时发现HIV感染，15例（23.8％）患者感染HIV到确诊淋巴瘤的时间间隔<1年，11例（17.5％）患者感染HIV到确诊淋巴瘤的时间间隔≥1年，12例（19.0％）患者时间间隔不详。42例（66.7％）患者进行了规范的抗HIV治疗，3例（4.8％）患者间断行抗HIV治疗，10例（15.9％）患者未行抗病毒治疗，8例（12.7％）患者情况不详。患者的主要临床表现为淋巴结肿大和腹痛，分别为37例（58.7％）和12例（19.1％），其他非典型症状包括纳差、乏力、吞咽困难、胸闷、咳嗽、鼻塞等。29例（46.0％）患者合并淋巴结外病变，17例（27.0％）患者合并大包块（最大直径≥7.5 cm），20例（31.7％）患者伴B症状。所有患者病理诊断均明确，其中生发中心来源（GCB）47例（74.6％），非GCB（non-GCB）16例（25.4％）。25例（39.7％）患者IPI评分0～2分，33例（52.4％）患者IPI评分3～5分，5例（7.9％）患者IPI评分不详。50例（79.4％）患者美国东部肿瘤协作组体能状况（ECOG-PS）评分0～1分，13例（20.6％）患者ECOG-PS评分2～4分。在可分期的59例患者中，Ann Arbor分期Ⅲ/Ⅳ期46例（78％），Ⅰ/Ⅱ期13例（22％）。29例（46.0％）患者有结外侵犯，其中11例为骨髓侵犯，3例为中枢神经系统侵犯，14例为胃肠道侵犯，5例为肝脏侵犯，2例为脾脏侵犯。63例HIV相关DLBCL患者中16例（25.4％）患者未接受化疗，14例（22.2％）患者接受小于4个周期的全身化疗，33例（52.4％）患者曾接受4个周期及以上的全身化疗；47例接受全身化疗的患者中，29例（61.7％）应用R-CHOP方案，18例（38.3％）应用CHOP方案。

2. 实验室检查：中位WBC 4.18（2.35～9.21）×10^9^/L，42.4％（25/59）患者WBC下降（<4×10^9^/L）；中位淋巴细胞计数1.12（0.29～3.93）×10^9^/L，47.5％（28/59）患者淋巴细胞计数下降（<1×10^9^/L）；中位中性粒细胞计数2.45（0.83～6.62）×10^9^/L，27.1％（16/59）患者中性粒细胞计数下降（<2×10^9^/L）；中位PLT 188（29～412）×10^9^/L，10.2％（6/59）患者PLT下降（<100×10^9^/L）。41.2％（21/51）患者NK细胞计数下降（<150×10^6^/L），15.7％（8/51）患者NK细胞百分比下降（<7％）。51例患者行免疫功能检测，其中CD4^+^淋巴细胞中位数为203（4～1022）×10^6^/L，25例（49％）患者CD4^+^细胞数<200×10^6^/L，26例（51％）患者CD4^+^细胞数≥200×10^6^/L。84.3％（43/51）患者CD4/CD8下降（<0.7），18.9％（10/53）患者CD5阳性。24.6％（14/57）患者Ki-67阳性率<80％，75.4％（43/57）患者Ki-67阳性率≥80％。18.9％（7/37）患者c-Myc阳性率<30％，62.2％（23/37）患者c-Myc阳性率≥30％，18.9％（7/37）患者c-Myc阴性。6.0％（3/50）患者BCL-2阳性率<30％，48.0％（24/50）患者BCL-2阳性率≥30％，46.0％（23/50）患者BCL-2阴性。血β_2_-微球蛋白（β_2_-MG）升高发生率91.1％（51/56），乳酸脱氢酶（LDH）升高发生率为81％（51/63）。58.1％（25/43）患者EB病毒DNA（EBV-DNA）<5×10^3^拷贝/ml，41.9％（18/43）患者EBV-DNA≥5×10^3^拷贝/ml。乙型肝炎表面抗原阳性发生率为12.7％（8/63），丙型肝炎IgG抗体阳性发生率为3.2％（2/63）。

3. 治疗方案：63例患者中，42例（66.7％）患者在感染科医师评估下行规范持续联合抗逆转录病毒治疗（cART，combination antiretroviral therapy），3例（4.8％）患者间断行cART，10例（15.9％）患者未行cART，8例（12.7％）患者是否行cART不详。16例患者未行全身化疗，其中10例未行任何治疗，均在14个月内死亡，中位生存时间3（1～14）个月，6例死于继发感染，3例死于淋巴瘤进展，1例死于冠心病。余6例患者仅行cART，其中3例继发感染死亡，2例失访，1例仍存活。在行全身化疗的47例患者中，29例（61.7％）患者采用R-CHOP样方案，具体为：利妥昔单抗375 mg·m^−2^·d^−1^，第0天；阿霉素50 mg·m^−2^·d^−1^，第1天；环磷酰胺750 mg·m^−2^·d^−1^，第1天；长春新碱1.4 mg·m^−2^·d^−1^，第1天；泼尼松60 mg·m^−2^·d^−1^，第1～5天；18例（38.3％）患者采用CHOP样方案，具体为：阿霉素50 mg·m^−2^·d^−1^，第1天；环磷酰胺750 mg·m^−2^·d^−1^，第1天；长春新碱1.4 mg·m^−2^·d^−1^，第1天；泼尼松60 mg·m^−2^·d^−1^，第1～5天。

4. 治疗疗效和总体生存：在可评估疗效的43例患者中，17例（39.53％）患者获得CR，20例（46.51％）患者获得PR，1例（2.33％）患者SD，5例（11.63％）患者PD。63例患者中7例失访，中位随访时间11（1～161）个月，死亡25例，56例患者的中位PFS时间和OS时间分别为14个月和36个月；1、2、3、5年PFS率分别为50.0％、44.4％、40.7％、36.7％，1、2、3、5年OS率分别为65.0％、53.8％、47.1％、43.5％。

5. 预后因素：对56例患者PFS和OS的预后因素进行单因素分析（表1）。PFS相关不良预后因素为：ECOG-PS评分2～4分（*P*＝0.049）、IPI评分3～5分（*P*<0.001）、β_2_-MG升高（*P*＝0.049）和全身化疗周期数<4次（*P*＝0.001）。OS相关不良预后因素为：年龄≥60岁（*P*＝0.012）、ECOG-PS评分2～4分（*P*＝0.043）、IPI评分3～5分（*P*＝0.001）、β_2_-MG升高（*P*＝0.007）和全身化疗周期数<4次（*P*<0.001）。多因素分析显示IPI评分3～5分、ECOG-PS评分2～4分和化疗周期数<4次是影响患者PFS的独立危险因素，年龄≥60岁、IPI评分3～5分、ECOG-PS评分2～4分和化疗周期数<4次是影响患者OS的独立危险因素（表2）。

**表1:** 56例HIV相关DLBCL患者2年PFS和2年OS相关变量单因素分析结果

因素	例数	PFS			OS		
		2年PFS率（％，*x*±*s*）	*χ*^2^值	*P*值	2年OS率（％，*x*±*s*）	*χ*^2^值	*P*值
性别			0.062	0.804		0.827	0.363
男	50	22.0±5.9			34.0±6.7		
女	6	16.7±15.2			16.7±15.2		
年龄（岁）			3.251	0.071		6.328	0.012
<60	42	23.8±6.6			38.1±7.5		
≥60	14	7.1±6.9			14.3±9.4		
病理类型			0.053	0.818		0.141	0.707
GCB	41	24.4±6.7			34.1±7.4		
non-GCB	15	13.3±8.8			26.7±11.4		
Ann Arbor分期			0.066	0.797		0.144	0.704
Ⅰ/Ⅱ	12	25.0±12.5			25.0±12.5		
Ⅲ/Ⅳ	41	22.0±6.5			31.7±7.3		
IPI评分（分）			13.558	<0.001		10.877	0.001
0～2	24	37.5±9.9			54.2±10.2		
3～5	29	3.4±3.4			10.3±5.7		
B症状			0.666	0.414		0.508	0.476
无	40	27.5±7.1			40.0±7.7		
有	14	7.1±6.9			14.3±9.4		
ECOG-PS评分			3.880	0.049		4.097	0.043
0～1分	46	26.1±6.5			34.8±7.0		
2～4分	10	0			10.0±9.5		
CD5			2.560	0.110		2.293	0.130
阴性	40	20.0±6.3			27.5±7.1		
阳性	9	33.3±15.7			44.4±16.6		
EB病毒DNA（拷贝/ml）			0.021	0.884		0.172	0.678
<5×10^3^	25	20.0±8.0			28.0±9.0		
≥5×10^3^	17	23.5±10.3			23.5±10.3		
CD4^+^细胞数（×10^6^/L）			1.175	0.278		2.991	0.084
<200	24	12.5±6.8			12.5±6.8		
≥200	25	28.0±9.0			40.0±9.8		
β_2_-微球蛋白			3.884	0.049		7.176	0.007
正常	5	60.0±21.9			80.0±17.9		
升高	46	15.2±5.3			21.7±6.1		
LDH			0.008	0.929		0.071	0.790
正常	10	10.0±9.5			20.0±12.6		
升高	46	23.9±6.3			34.8±7.0		
淋巴结外受累			2.847	0.092		0.537	0.464
否	31	25.8±7.9			35.5±8.6		
是	25	16.0±7.3			28.0±9.0		
骨髓受累			3.467	0.063		0.133	0.716
否	46	28.3±6.6			34.8±7.0		
是	10	0			10.0±9.5		
大包块（≥7.5cm）			0.461	0.497		1.957	0.162
否	41	22.0±6.5			34.1±7.4		
是	15	20.0±10.3			20.0±10.3		
化疗周期数			14.436	0.001		26.901	<0.001
0	13	0			0		
<4	11	9.1±8.7			9.1±8.7		
≥4	32	34.4±8.4			50.0±8.8		
是否使用利妥昔单抗			2.591	0.107		3.735	0.053
否	15	21.4±7.8			28.6±8.5		
是	28	33.3±12.2			53.3±12.9		

注：DLBCL：弥漫大B细胞淋巴瘤；PFS：无进展生存；OS：总生存；GCB：生发中心来源；non-GCB：非生发中心来源；B症状：发热、盗汗、体重减轻；ECOG-PS：美国东部肿瘤协作组体能状况

**表2:** 56例HIV相关DLBCL患者无进展生存（PFS）和总生存（OS）相关变量多因素分析结果

因素	PFS		OS	
	*HR*（95％ *CI*）	*P*值	*HR*（95％ *CI*）	*P*值
年龄（≥60岁，<60岁）	1.990（0.990～4.001）	0.053	2.272（1.110～4.651）	0.025
IPI评分（3～5分，0～2分）	2.830（1.468～5.457）	0.002	3.562（1.794～7.074）	<0.001
ECOG-PS评分（2～4分，0～1分）	2.347（1.036～5.314）	0.041	2.675（1.162～6.153）	0.021
β_2_-微球蛋白（升高，正常）	1.698（0.618～4.669）	0.305	2.212（0.793～6.170）	0.129
化疗周期数（≥4，<4）	0.418（0.266～0.656）	<0.001	0.290（0.176～0.479）	<0.001

注：DLBCL：弥漫大B细胞淋巴瘤；ECOG-PS：美国东部肿瘤协作组体能状况

## 讨论

20世纪90年代以来，随着cART的广泛应用，HIV感染者预期寿命得到了显著延长，一项来自丹麦的大型队列研究对5 701例HIV感染者和28 505名配对的健康人进行随访，中位死亡年龄从1995-1996年的34.5岁提高到2010-2015年的73.9岁[5]。截至2019年12月31日，我国共报告艾滋病患者58万余例，其中42万余例生存，2007至2019年死亡比例从10.9％下降到4.3％[6]。在HIV感染人群中，HIV相关NHL发病率为（100～300）/10万，超过KS，成为最常见的HIV相关肿瘤[7]。HIV相关NHL中95％以上为B细胞来源，最常见的病理类型为DLBCL，确诊时多为Ⅲ/Ⅳ期，肿块短期内快速生长，常伴随B症状及结外受累[8]。本研究63例HIV相关DLBCL患者中，46.0％的患者合并淋巴结外病变，结外病变多见于骨髓和胃肠道；31.7％的患者伴随B症状。在可分期的59例患者中，Ann Arbor分期多为Ⅲ/Ⅳ期，占78％，与上述文献结果类似。63例HIV相关DLBCL患者，男57例（90.5％），女6例（9.5％），诊断时中位年龄49（23～87）岁，其中48例（71.2％）<60岁，与我国总体艾滋病患者的性别和年龄类似[6]。病理类型方面，HIV相关DLBCL中GCB亚型更常见[9]，本研究中GCB亚型占74.6％（47/63）。

HIV导致DLBCL高发的发病机制尚不明确，多项研究显示，HIV感染机体后导致细胞免疫功能受损，一些致肿瘤性病毒，如人类疱疹病毒8型（HHV-8）、EBV、乙型肝炎病毒（HBV）和丙型肝炎病毒（HCV）等伺机感染机体，激活B淋巴细胞系统，慢性抗原持续刺激及细胞因子分泌失调等导致DLBCL发生，但具体机制尚未得到阐释[10]–[12]。

目前HIV感染相关DLBCL的治疗国内外尚无统一标准，通常采用与HIV阴性患者相同的治疗方案及cART。随着cART的广泛应用，HIV相关DLBCL患者的生存亦有显著提升。一项来自法国的前瞻性队列研究纳入2008-2015年间22个医疗中心的110例HIV感染相关NHL患者，其中DLBCL 52例（47％），大部分结外起病（81％），ECOG-PS评分>1分者18例（35％），年龄调整的国际预后指数（aaIPI）2～3分29例（58％），44例（85％）患者接受R-CHOP方案化疗，生存分析显示，患者的2年OS率和PFS率均为75％[13]。在cART时代，加入利妥昔单抗的R-CHOP方案可使HIV相关DLBCL患者的2年OS率达到75％，与同期、同aaIPI评分的HIV阴性DLBCL患者生存相似。孙开宇等[14]回顾性分析53例HIV感染相关NHL患者，其中DLBCL占60.4％（32/53），仅20例患者接受全身化疗，患者的1、2、3、5 年 OS 率分别为39.6％、32.7％、27.7％和20.1％。Wu等[15]回顾性分析2012年6月至2018年6月诊断的100例HIV相关淋巴瘤患者，其中DLBCL 66例，采用CHOP/EPOCH样方案化疗。接受全身化疗的85例患者2年OS率为41.0％，中位PFS时间为4个月。本研究63例患者中7例失访，43例（68.3％）患者接受全身化疗，其中65.1％（28/43）的患者采用R-CHOP样方案，34.8％（15/43）的患者采用CHOP样方案，化疗周期数<4的患者占25.6％（11/43）。中位随访11（1～161）个月，死亡25例，56例患者的中位PFS时间和OS时间分别为14个月和36个月，1、2、3和5年OS率分别为65.0％、53.8％、47.1％和43.5％。与采用CHOP样方案化疗的患者相比，接受R-CHOP样方案化疗的患者OS时间有延长的趋势（*P*＝0.053）。本研究患者的总体生存略优于国内同期水平，但低于国外报道水平，可能与患者放弃治疗比例较高、未普遍使用CD20单抗有关。

Besson等[13]分析与HIV感染相关NHL患者的预后相关因素发现，ECOG-PS评分>1分（*HR*＝3.3，95％*CI* 1.2～8.9，*P*<0.05）、大于一处结外病变（*HR*＝3.4，95％*CI* 1.1～10.5，*P*<0.05）和aaIPI评分2～3分（*HR*＝3.7，95％*CI* 1.0～13.1，*P*<0.05）为影响OS的不良预后因素。Wu等[15]对HIV感染相关NHL患者预后相关因素分析发现，高aaIPI评分（*HR*＝11.92，95％*CI* 2.76～51.52，*P*＝0.001）、LDH升高（*HR*＝2.32，95％*CI* 1.09～4.92，*P*＝0.029）和未行全身化疗（*HR*＝0.16，95％*CI* 0.06～0.39，*P*<0.001）是影响OS的不良预后因素。Wu等[12]研究发现ECOG-PS评分>1分（*HR*＝10.54，95％*CI* 4.45～24.96，*P*＝0.003）和IPI评分3～5分（*HR*＝6.30，95％ *CI* 2.74～14.47，*P*＝0.004）是影响HIV感染相关DLBCL患者预后的不良因素。本研究发现与HIV感染相关DLBCL患者OS相关的不良预后因素为：年龄≥60岁（*P*＝0.012）、ECOG-PS评分2～4分（*P*＝0.043）、IPI评分3～5分（*P*＝0.001）、β_2_-MG升高（*P*＝0.007）和全身化疗周期数小于4次（*P*<0.001）。多因素分析发现年龄≥60岁，IPI评分3～5分，ECOG-PS评分2～4分和化疗周期数<4次是影响OS的独立危险因素。本研究预后相关因素与文献[12]–[13], [15]基本符合，提示在cART时代，影响HIV相关DLBCL患者生存的因素主要来自淋巴瘤本身，而非HIV感染。

综上，HIV相关DLBCL是最常见的HIV相关肿瘤，男性发病率高于女性，确诊时多为Ⅲ/Ⅳ期，肿块短期内快速生长，常伴随结外受累。cART联合全身化疗可显著提高患者生存率。本组患者放弃治疗比例及失访率较高，在未来临床工作中需重视并加强规范化诊疗和全程管理。
